# Knowledge as prevention: A cost‐effective intervention to reduce prenatal alcohol exposure

**DOI:** 10.1111/acer.70089

**Published:** 2025-08-01

**Authors:** Orlagh Keating, Ruth H. Brown, Stewart McDougall, Renate Kuenssberg, Suzanne O'Rourke

**Affiliations:** ^1^ Clinical and Health Psychology Department University of Edinburgh Edinburgh UK; ^2^ NHS Fife Psychology Department Lynebank Hospital Dunfermline UK

**Keywords:** alcohol, attitudes toward drinking, educational intervention, knowledge, pregnancy

## Abstract

**Background:**

Prenatal alcohol exposure (PAE) remains high despite international guidelines recommending alcohol abstinence during pregnancy. This poses a significant public health concern, as PAE is known to have harmful effects on fetal development and is the cause of fetal alcohol spectrum disorder (FASD). Enhancing knowledge and shifting attitudes toward PAE may help reduce its occurrence, but evidence remains limited. This study examined the impact of an educational intervention on knowledge and attitudes toward PAE among pregnant and recently pregnant women.

**Methods:**

A total of 1536 UK‐based women aged 19–50 years (*M* = 33.3 years) participated in an anonymous online questionnaire with an embedded intervention consisting of an existing information leaflet, “Alcohol and Pregnancy,” developed by the Royal College of Obstetricians and Gynaecologists. Participants completed pre‐ and postintervention evaluations of their knowledge and attitudes toward PAE.

**Results:**

The intervention led to significantly increased negative attitudes toward PAE (*z* = −9.67, *p* < 0.001, *r* = 0.29) and improved knowledge of its associated risks (*z* = −21.16, *p* < 0.001, *r* = 0.65). Regression analysis indicated that participants with alcohol‐exposed pregnancies, and with more positive initial attitudes and lower baseline knowledge of PAE‐associated risks, experienced the greatest changes postintervention (*F* [11, 1021] = 25.42, *p* < 0.001, adj. *R* ^2^ = 0.208).

**Conclusion:**

These findings highlight the effectiveness of low‐cost, self‐administered educational interventions in enhancing knowledge of risks and discouraging alcohol use during pregnancy. Implementation of such interventions in community, clinical, and online settings is recommended to reduce prenatal alcohol consumption.

## Graphical abstract

Changing knowledge and attitudes toward prenatal alcohol exposure (PAE) may aid in reducing PAE prevalence. The feasibility of an educational intervention, improving knowledge and attitudes toward PAE, was evaluated in a sample of 1536 pregnant, or recently pregnant, women. Comparisons of pre‐ and postintervention knowledge and attitudes revealed significant improvement in both factors. The intervention was most effective in those with alcohol‐exposed pregnancies and those with initially less informed attitudes and lower baseline knowledge of PAE‐associated risks.

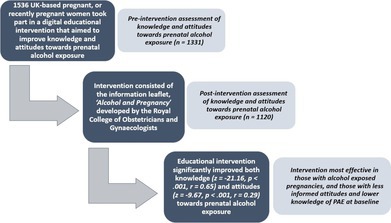

## INTRODUCTION

Prenatal alcohol exposure (PAE) remains a public health concern, despite international guidelines endorsing alcohol abstinence throughout pregnancy (World Health Organization, 2018). Globally, the prevalence of PAE is estimated at 9.8%, with rates exceeding 40% in some European countries (Popova et al., 2017). The negative effects of PAE on fetal development are well documented, including increased risk of miscarriage, preterm birth, perinatal mortality, and fetal alcohol spectrum disorder (FASD). FASD is an umbrella term encompassing a range of developmental differences resulting from fetal alcohol exposure, including central nervous system abnormalities; cognitive, physical, and neurobehavioral deficits; and sentinel facial features (SIGN, 2019). However, due to the complexities associated with PAE screening and FASD diagnosis (Bakhireva et al., 2018), accurate prevalence rates remain uncertain. In the UK, FASD prevalence has conservatively been estimated as 32.4 per 1000 population (Popova et al., 2017). A more recent UK population‐based cohort study provided a retrospective screening prevalence estimate of the condition, finding an FASD prevalence of 6%, which increased to 17% when missing data were accounted for using multiple imputation (McQuire et al., 2019).

Three broad approaches have been adopted to reduce PAE. Universal prevention strategies aim to raise awareness of the risks associated with alcohol use during pregnancy and influence public attitudes through public health initiatives, such as media campaigns and educational programs (e.g., Margan, 2022; Reynolds et al., 2021). Selective prevention strategies target women of reproductive age and at‐risk populations, such as those who consume alcohol during pregnancy, and include clinical interventions, such as brief interventions and motivational interviewing (e.g., Popova et al., 2023). Indicated prevention strategies focus on women who are at high risk, such as those requiring referral to specialist alcohol services, and may incorporate pharmacological interventions (Clarren et al., 2011).

Among these approaches, universal and selective prevention strategies have been most widely implemented. Reviews of randomized and nonrandomized controlled trials in pregnant populations support the effectiveness of universal prevention strategies in increasing knowledge and reducing PAE (Crawford‐Williams et al., 2015) and selective prevention strategies in improving abstinence rates (Gilinsky et al., 2011; Popova et al., 2023; Stade et al., 2009; Ujhelyi Gomez et al., 2021). However, these studies reported considerable methodological heterogeneity. Despite this, authors concluded that there was evidence to suggest that brief interventions are beneficial in supporting women to maintain abstinence during pregnancy. Moreover, brief interventions have been found to significantly reduce “special occasion” drinking during pregnancy (Tsang et al., 2022), and improve newborn health outcomes compared with control groups (O'Connor & Whaley, 2007). Brief interventions have therefore been recommended as cost‐effective preventive measures that have robust evidence‐bases (O'Donnell et al., 2014). These interventions typically include motivational interviewing, goal setting, and assessment for alcohol dependency (e.g., Project CHOICES Intervention Research Group, 2003). For women at high risk, interventions involve screening for hazardous drinking and structured conversations aimed at supporting behavioral change to reduce harm (Scobie & Woodman, 2017).

Brief educational and psychological interventions show most promise as they are equitable, easily accessible, quick to deliver, and have low implementation costs (e.g., Popova et al., 2023; Scobie & Woodman, 2017). The use of web‐based interventions for alcohol misuse has also grown in recent years, offering a cost‐effective mode of delivery (Balhara & Verma, 2014). While these interventions have been primarily utilized in youth populations and, to a lesser extent, in pregnant women known to misuse alcohol (e.g., Martinez‐Montilla et al., 2020; Tenkku et al., 2011, respectively), they have not yet been widely explored in pregnant women without a history of alcohol misuse. Further research is therefore needed to evaluate the use of educational interventions in these broader populations.

Providing clear information on the risks associated with PAE and current guidelines may help shift attitudes and influence social norms surrounding alcohol consumption during pregnancy (British Medical Association, 2016). This is supported by research on social marketing interventions (Kubacki et al., 2015) and studies demonstrating that universal interventions aimed at raising awareness of PAE can positively impact attitudes (e.g., Pettigrew et al., 2023), thus having the potential to reduce the prevalence of FASD (Chersich et al., 2012). Educational interventions and contact with individuals with a stigmatized condition may assist in reducing stigma (Roozen et al., 2020). Attitudes toward PAE and drinking behaviors during current and past pregnancies have been found to be the strongest predictors of PAE (Peadon et al., 2010, 2011); however, research in this area remains limited.

A lack of knowledge regarding the risks of PAE may further increase the likelihood of PAE. For instance, qualitative research on women's perceptions of the information and advice given around PAE highlights ongoing confusion about safe consumption levels and inconsistent messaging (e.g., Latuskie et al., 2019), likely exacerbated by changes in guidance over time. Similar concerns have been echoed in UK‐based studies of pregnant women (Frennesson et al., 2024; Grant et al., 2019). However, research from countries with well‐established PAE awareness campaigns, school‐based education programs and media initiatives (e.g., Australia), has shown high levels of public awareness, with 92.7% of women acknowledging alcohol's harmful effect on fetal development (*n* = 1103; Peadon et al., 2010).

Overall, knowledge and attitudes are key determinants of health behavior, and within the context of PAE, improving these factors may help reduce the risk of alcohol‐exposed pregnancies (e.g., Reynolds et al., 2021). Historically, research evaluating PAE prevention efforts has focused on changes in maternal drinking behavior as a measure of intervention effectiveness (Ospina et al., 2011), and has primarily recruited women who either reported PAE or were considered high risk (Gilinsky et al., 2011; Stade et al., 2009; Ujhelyi Gomez et al., 2021), limiting generalizability to the wider population. To date, only one education intervention in the UK has targeted a general, rather than pregnant, population (Reynolds et al., 2021).

This study builds on existing evidence supporting the effectiveness of brief educational interventions and the role of knowledge and attitudes in influencing PAE. Specifically, we assess the impact of an inexpensive, self‐administered intervention, comprising an information leaflet, on changing attitudes and knowledge toward PAE in a sample of pregnant, or recently pregnant, women. Additionally, we evaluate which factors emerge as significant predictors of knowledge and attitudes toward PAE.

## METHOD

This cross‐sectional study implemented a quasi‐experimental, within‐subjects, pre‐ and postintervention design. An online, self‐administered, leaflet intervention and anonymous questionnaire were administered via Qualtrics (2005; https://www.qualtrics.com).

### Participants

Inclusion criteria were women in the UK over the age of 18, who had been pregnant at any time since April 1, 2016 or were pregnant at the time of participation (during the recruitment period July 20, 2020 to December 31, 2020). Participants had to be residing in the UK at the time of pregnancy and study completion and have sufficient English language literacy skills to complete the survey. The date of April 1, 2016 was chosen as a cutoff point due to changes in policy and healthcare guidance on PAE in the UK. There was no restriction placed on whether it was the participants' first pregnancy, and participants were advised to report on their most recent pregnancy if they had had more than one since April 2016.

### Materials

#### Demographic information

A bespoke questionnaire gathered information on participants' age, ethnicity, marital status, employment status, level of education, and mental health status. Information on pregnancy was also captured, including the year of pregnancy, location of residency at the time, whether it was a planned pregnancy, and at what stage the participant found out they were pregnant.

#### Alcohol Use Disorders Identification Test—Consumption (AUDIT‐C; Bush et al., 1998)

Information of PAE was collected via the Alcohol Use Disorders Identification Test—Consumption (AUDIT‐C; Bush et al., 1998); previously validated for use in pregnant populations (Dawson et al., 2005); and recommended for guiding conversation on alcohol use during pregnancy (Murdoch Children's Research Institute, 2010). It has robust psychometric properties and has been shown to demonstrate high levels of sensitivity (95%) and specificity (85%) in pregnant women (Burns et al., 2010). One item from the AUDIT‐C was used to identify alcohol‐exposed pregnancies in the sample; “During your pregnancy, how often did you have a drink containing alcohol?”. Participants could respond either “never” “monthly or less,” “2–4 times per month,” or “2–3 times per week.”

#### Alcohol and Pregnancy Questionnaire (APQ; Peadon et al., 2011)

##### Attitudes

Attitudes and knowledge of PAE were assessed utilizing a 12‐item Alcohol and Pregnancy Questionnaire (“APQ”; Peadon et al., 2011). The measure was initially adapted from the Health Canada survey titled “Alcohol Use During Pregnancy and Awareness of Fetal Alcohol Syndrome” (Environics Research Group, 2006) and remains the only psychometric measure developed to capture attitudes and knowledge toward PAE. Participants responded to these items (e.g., on a 5‐point Likert scale [from 1 = “Strongly agree” to 5 = “Strongly disagree”]). Higher scores are indicative of more positive attitudes toward PAE.

##### Knowledge

Participants were asked three initial knowledge items; if they were aware of FASD (“Have you heard of the term FASD?”), if they were aware of the guidelines and recommendations from the UK's CMO regarding alcohol consumption during pregnancy (“Were you aware of this guidance?”), and if they believed that the CMO message was known to the general population (“There is no safe amount of alcohol during pregnancy. Do you feel this message is widely known throughout the UK?”). All initial questions were rated “yes,” “maybe,” or “no.”

Participants then rated six items on knowledge of the risks associated with PAE, such as miscarriage and seizures using the APQ. A further two questions were designed and added by the research team, “Please tell us how much you agree with the guidance that there is no safe level of alcohol use during pregnancy,” and “Please tell us how much you agree with the guidance that women should avoid alcohol completely during pregnancy.” Participants were asked to rate these items on a 5‐point Likert scale, with 1 = “Strongly agree” to 5 = “Strongly disagree.” Higher scores are indicative of less knowledge of PAE.

#### Educational intervention leaflet

The intervention comprised an information leaflet known as “Alcohol and pregnancy”, developed by the Royal College of Obstetricians and Gynaecologists (2018) (accessible at: https://www.yorkshirebabyscan.co.uk/uploads/pi‐alcohol‐and‐pregnancy.pdf). The leaflet outlined the effects of prenatal alcohol consumption on fetal development, gave information about FASD and guidance on alcohol consumption during pregnancy.

### Procedure

Recruitment took place online via social media platforms, such as Facebook, Twitter, and LinkedIn. The study was advertised via an online poster and promoted on various sites on these platforms, for example Facebook “Mums” and “Mums‐to‐be” groups. Organizations, including Mumsnet, National Childbirth Trust, and Maternal Mental Health Alliance, were invited to share details on their webpages, forums, and social media platforms. Emails with the poster were sent to the above organizations monthly during the recruitment phase, which provided a description of the study and a link to the study on Qualtrics. The Qualtrics link led first to the participant information sheet and then to the consent form. Consenting participants were asked to provide their demographic information and complete the preintervention iteration of the APQ, and then to read the information leaflet. As part of the wider research project, reported elsewhere, participants were also asked to complete a measure of their alcohol use before and during pregnancy, and their behavioral activation and inhibition. Lastly, participants were asked to complete the postintervention iteration of the APQ. On completion, a debrief page was provided. The study took approximately 20 min, and participants were given the option to enter a prize draw to win one of three £50 Amazon gift cards on completion of the questionnaire. No identifiable participant information was collected in the survey. Ethics approval was obtained by the University of Edinburgh School of Heath in Social Sciences Ethics Committee.

### Data analysis

Descriptive analysis of the sample was first completed with the demographic variables listed above. A series of Wilcoxon signed‐rank tests were then performed to assess the efficacy of the intervention on attitudes and knowledge toward PAE. Effect sizes, as determined by Cohen's *r* (Cohen, 1988), were calculated, following the guidance of Fritz et al. (2012). Cohen's *r* values between 0 and 0.29 were considered small effect sizes, 0.30–0.49 were considered medium effect sizes, and >0.50 were considered large effect sizes (Cohen, 1988). Subsequently, attitude and knowledge scores were amalgamated, and a multiple regression analysis was conducted to assess the factor(s) predictive of score changes in attitudes and knowledge postintervention. Predictive variables were age, level of education, marital status, year of pregnancy, pregnancy planning, knowledge of CMO guidance on PAE, alcohol‐exposed pregnancy, and preattitude and knowledge scores. These were re‐categorized (shown in Table 1) and dummy variables created for age (19–25; 26–35; and 36–50) level of education (secondary education level [e.g., standard grades], undergraduate degree [i.e., Bachelor's Degree], postgraduate degree [e.g., doctorate], and other qualification [e.g., vocational training], and year of pregnancy [2016–2019; and currently pregnant at time of study participation]). Moreover, marital status (partner/no partner); alcohol‐exposed pregnancy (exposed/not exposed); and awareness of CMO guidelines (aware/not aware) were dichotomized and dummy coded. Lastly, combined preintervention attitude and knowledge scores were inserted into the model as a continuous variable. Reference categories within the categorical predictive variables were selected based on the category with the largest sample size. All statistical analyses were conducted using IBM SPSS Statistics (2017, Version 25).

**TABLE 1:** Participant demographic information.

Characteristic	*N*	%
Age (*N* = 1456)		
19–25	90	6.2
26–35	895	61.5
36–50	471	32.3
Marital/relationship status (*N* = 1412)		
With partner	1317	93.7
No partner	95	6.3
Highest qualification received (*N* = 1463)		
Secondary education (i.e., Standard grades/GCSE level or higher)	238	16.3
Undergraduate degree (i.e., Bachelor's)	516	31.6
Postgraduate degree (i.e., Master's Degree, Doctorate, Postgraduate Certificate)	554	37.9
Trade/vocation/other	155	14.2
Year of pregnancy (*N* = 1467)		
2016–2019	1020	69.5
Currently pregnant (2020)	447	30.5
Pregnancy planning (*N* = 1467)		
Planned	1214	82.8
Unplanned	253	17.2
Alcohol‐exposed pregnancies (*N* = 1355)		
Exposed	342	25.2
Not exposed	1013	74.8

### Data distribution

Data were first screened to test that it met statistical assumptions. Due to the ordinal nature of the data, Wilcoxon signed‐rank tests were conducted to determine the efficacy of the intervention. Cases with missing data were omitted and analyses were conducted for full datasets only. Difference scores were approximately symmetrically distributed, as indicated by a Shapiro–Wilk statistic of *p* = 0.94. Tests of skewness and kurtosis indicated that the data were not normally distributed for both pre‐ and postintervention scores on the attitude and knowledge measure (*z* = 11.95 and *z* = 14.69, respectively). This was also observed by Shapiro–Wilk's test for normal distribution (*p* < 0.05). However, research indicates that larger sample sizes are likely to be overly sensitive to statistical tests of deviation, and minor deviations can produce significant *p* values (Uttley, 2019). Given the *W* statistic was large for both pre‐ and postattitude data (*p* = 0.94 and *p* = 0.92 respectively), the deviation was minor and Wilcoxon signed‐rank tests were therefore completed. Assumptions for parametric tests were met for multiple regression; independence of residuals was indicated by a Durbin–Watson statistic of 1.94, there was no evidence of multicollinearity as observed by tolerance values greater than 0.1 and variance inflation factor values lower than 5. Values for Cook's distance above 1, and visual inspection of a *Q*–*Q* plot, indicated normality. Histograms and boxplots highlighted a violation of the assumption of outliers. There were 13 outliers, as indicated by standardized residual values greater than 3, which were removed prior to statistical analysis.

## RESULTS

### Participant demographics

Overall, 1663 participants followed the Qualtrics link and 1536 (92%) provided consent. The typical respondent was 33.3 years of age (SD = 4.89); white (98%); married or cohabiting with a partner (92.4%); and had completed a bachelor's degree or higher (73%) (see Table S1 for a full overview of the sample's demographic information). The distributions of knowledge and attitude scores, pre‐ and postintervention, can be seen in Table 2.

**TABLE 2:** Summary of knowledge and attitudes scores.

	*N*	Mean	SD	Score range	*α*
Preintervention					
Attitudes	1324	17.87	4.37	12–37	0.671
Knowledge	1294	14.53	4.71	8–31	0.822
Total	1286	32.42	8.27	20–64	0.844
Postintervention					
Attitudes	1108	16.94	3.91	12–33	0.628
Knowledge	1058	11.86	11.85	8–28	0.843
Total	1048	28.73	7.30	20–37	0.799

### Impact of educational intervention on attitudes toward PAE

A total of 1331 participants provided responses to the APQ prior to the intervention and 1120 postintervention, giving an attrition rate of 16% (*n* = 211) (see Table S2 for a full overview of group comparisons). Significant group differences emerged between those who did and did not complete the questionnaire postintervention on most demographic variables; however, this is likely due to the sensitivity of chi‐square analyses toward sample sizes (Bergh, 2015) rather than observable inter‐group differences.

**FIGURE 1:**
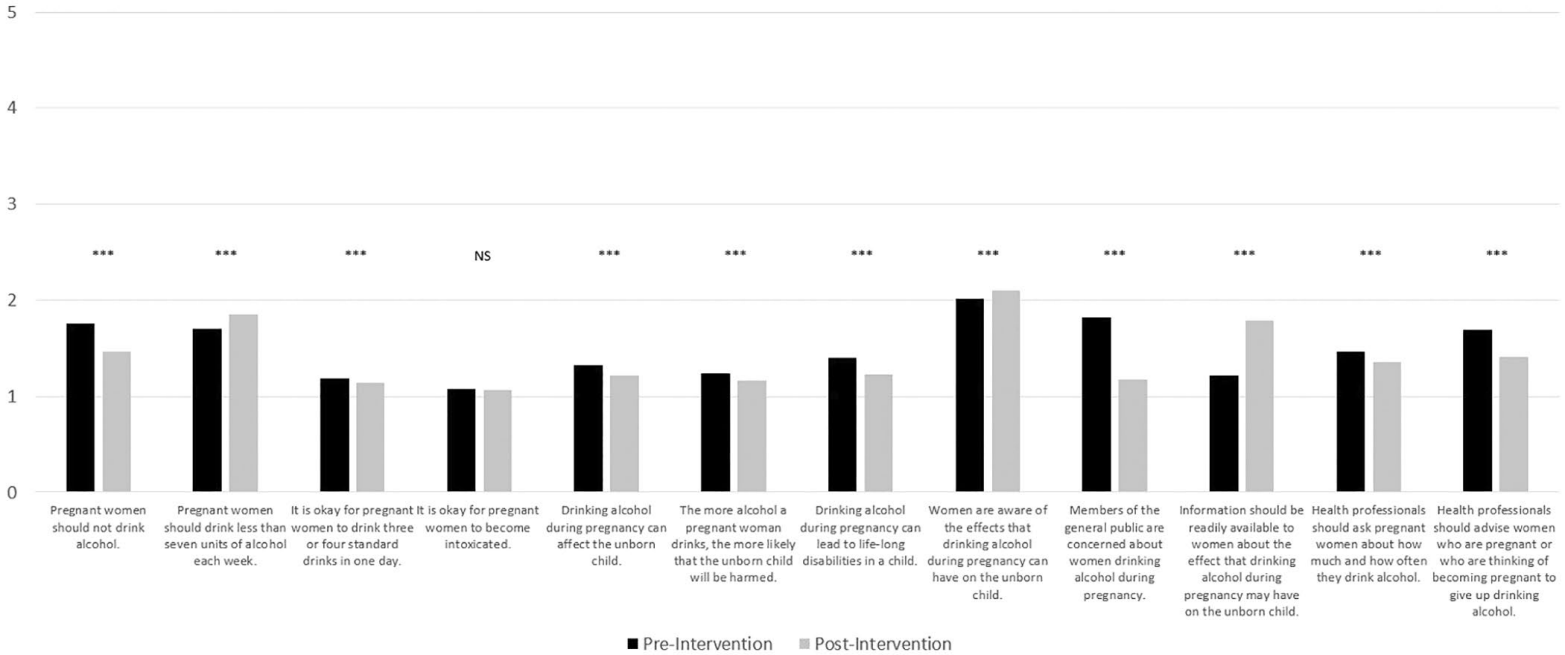
Results from Wilcoxon signed‐rank tests in attitude score change, pre‐ to postintervention. Lower scores are indicative of more negative attitudes toward PAU. NS, nonsignificant. ***p < 0.001.

To assess differences between pre‐ and postintervention attitude scores following the intervention, responses to all 12 statements were collated and scored to provide total scores and means (see Figure 1 and Table S3). APQ items 3 (“It is okay for pregnant women to drink three or four standard alcoholic drinks in one day”) and 4 (“It is okay for pregnant women to become intoxicated”) were reverse‐coded to ensure that all items were aligned in the same direction for consistent interpretation of total scores. Preintervention attitude scores ranged from 12 to 37 (mean score = 17.87, SD = 4.37) and postintervention attitude scores ranged from 12 to 33 (mean score = 16.94, SD = 3.91). At the total score level, Wilcoxon signed rank‐test determined that there was a statistically significant median decrease in positive attitudes toward drinking following the intervention (*Mdn* = 16) compared with attitudes prior to the intervention (*Mdn* = 17) with a small effect size (*z* = −9.67, *p* < 0.001 *r* = 0.29). At the individual item level, most items demonstrated a significant change in scoring postintervention, from small effect sizes (e.g., “It is okay for pregnant women to drink three or four standard drinks in one day”; *z* = −2.60, *p* < 0.001, *r* = 0.07) to large effect sizes (e.g., “Members of the general public are concerned about women drinking alcohol during pregnancy”; *z* = −19.90, *p* < 0.001, *r* = 0.59). One item, “It is okay for pregnant women to become intoxicated,” did not significantly change postintervention (*z* = 0.802, *p* < 0.001, *r* = 0.02).

### Impact of intervention on knowledge of risks of PAE and CMO guidance

**FIGURE 2:**
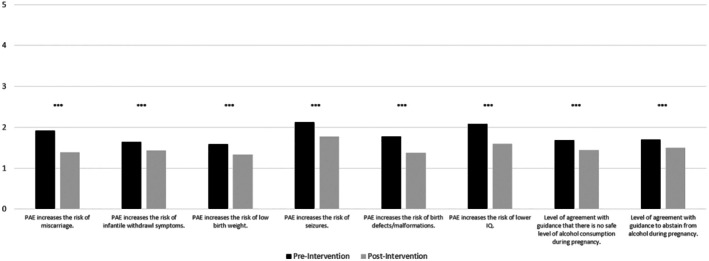
Results from Wilcoxon signed‐rank tests in knowledge score change, pre‐ to postintervention. Lower scores are indicative of increased knowledge. PAU, prenatal alcohol use. ***p < 0.001.

The majority of participants had heard of the term FASD and were aware of the CMO's guidance (85.5%, *n* = 1130). When asked to respond to the following item, “There is no safe amount of alcohol during pregnancy. Do you feel that this message is widely known throughout the UK?”, 37.2% of participants reported “no” (*n* = 491), 21.7% (*n* = 287) were unsure, and 41.1% of participants reported “yes” (*n* = 543). Several Wilcoxon signed‐rank tests were then conducted to determine differences in knowledge postintervention (see Figure 2 and Table S4).

A significant difference was observed between total pre‐ and postintervention ratings across items assessing both knowledge of risks associated with PAE and level of agreement with the UK CMO's guidance (*z* = −21.15, *p* < 0.001, *r* = 0.65). At the total knowledge score level, scores ranged from 8 to 31 preintervention (mean score = 14.53, SD = 4.71), and from 8 to 28 postintervention (mean score = 11.85, SD = 11.86), indicating a general increase in knowledge of both the CMO guidance and risks of PAE. Furthermore, at the individual item level, all knowledge questions showed significant differences indicating improved knowledge pre‐ and postintervention, with effect sizes ranging from medium (e.g., “Prenatal alcohol exposure increases the risk of infantile withdrawal symptoms”; *z* = −8.8, *p* < 0.001, *r* = 0.27) to large (e.g., “Prenatal alcohol exposure increases the risk of miscarriage”; *z* = −18.05, *p* < 0.001, *r* = 0.54).

### Factors predictive of change in knowledge and attitudes

A multiple regression was conducted to explore if participant demographic variables, awareness of the CMO guidance on alcohol abstinence during pregnancy, and preintervention attitudes and knowledge toward PAE predicted improvement in combined attitudes and knowledge scores following the intervention. Prior to the analysis, a change score was generated, whereby the combined postintervention attitude and knowledge scores were subtracted from the combined preintervention attitude and knowledge scores. In order to insert categorical variables into the model, demographic variables were dummy coded and reference categories were utilized for ease of result interpretation (see Table 3). The model predicted statistically significant change scores in attitude and knowledge with a medium effect (*F* [11, 1021] = 25.42, *p* < 0.001, adj. *R* ^2^ = 0.208). After inserting all of the predictive variables into the model, preintervention attitude and knowledge scores (*β* = −0.478, *p* < 0.001) and having an alcohol‐exposed pregnancy (*β* = 0.083, *p* = 0.009) emerged as the only significant predictors of change score, indicating that those who were both more tolerant toward PAE initially and who had lower knowledge of PAE risks were more likely to evidence a change in scores; and those who had an alcohol‐exposed pregnancy were more susceptible to attitude and knowledge change.

**TABLE 3:** Multiple regression results for change scores in attitudes and knowledge.

Predictive variable	*B*	95% CI for *B*		SE *B*	*β*	*R* ^2^	Adj. *R* ^2^
		LL	UL				
Constant	5.61	4.38	6.86	0.630		0.217	0.208***
Age (Reference category: 26–35)							
Age (19–25)	0.667	−0.553	1.88	0.622	0.031		
Age (36–50)	−0.163	−0.740	0.414	0.294	−0.016		
Year of pregnancy (Reference category: 2016–2019)							
Currently pregnant (2020)	0.510	−0.065	1.09	0.293	0.050		
Level of education (Reference category: Postgraduate degree)							
Secondary education	0.036	−0.803	0.875	0.428	0.003		
Bachelor's degree	−0.298	−0.903	0.307	0.308	−0.030		
Trade/vocational/other	−0.838	−1.75	0.076	0.466	−0.054		
Marital/relationship status (Reference category: With partner)							
No Partner	0.820	−0.349	1.99	0.596	0.041		
Pregnancy planning (Reference category: Planned pregnancy)							
Unplanned	0.172	−0.581	0.926	0.384	0.013		
Awareness of CMO guidance (Reference category: Aware)							
Unaware	−0.720	−1.47	0.033	0.384	−0.053		
Alcohol exposure during pregnancy (Reference category: No use)							
Exposed pregnancy	0.928	0.233	0.233	0.354	0.083**		
Preintervention knowledge and attitude scores	−0.262	−0.319	−0.246	0.017	−0.444***		

Abbreviations: Adj. *R* ^2^, adjusted *R* ^2^; *B*, unstandardized regression coefficient; CI, confidence interval; LL, lower limit; *R* ^2^, coefficient of determination; SE *B*, standard error of the coefficient; UL, upper limit; *β*, standardized coefficient.

**p < 0.01, ***p < 0.001.

## DISCUSSION

The current study expands upon the limited body of research, suggesting that educational interventions can positively influence knowledge and attitudes toward PAE. To the authors' knowledge, this is the first attempt to establish such findings in a sample of currently or recently pregnant women in the UK.

Preintervention, most women were found to already hold negative attitudes toward PAE and supported the UK's CMO's recommendation that pregnant women should abstain from alcohol. This aligns with both quantitative (Fletcher et al., 2023) and qualitative (Holland et al., 2016) research, which has demonstrated support for this abstinence message, though prior studies have also highlighted that endorsement of such a message does not necessarily translate into adherence (Holland et al., 2016). The majority of women disagreed that it was acceptable for pregnant women to become intoxicated and concurred that PAE carried risks to the fetus, consistent with findings from Esposito et al. (2015). Additionally, most women agreed that healthcare professionals should inquire about alcohol consumption during pregnancy.

Despite broad recognition of the risks associated with PAE, specific knowledge about FASD or the UK CMO's guidance remained inconsistent. In this study, 82% of women had heard of FASD; less than that observed in an older Canadian survey where 88% had heard of the condition (Environics Research Group, 2006). Furthermore, 15% were unaware of the UK CMO's guidance on PAE, and over one‐third (35.8%) felt that the abstinence message was not widely known across the UK. These findings suggest that the CMO's recommendation of abstinence, introduced prior to participant's pregnancies, is not effectively reaching prospective mothers. This highlights an ongoing need for stronger public health messaging to improve awareness and understanding of PAE risks.

Despite the majority of participating women initially holding negative attitudes toward PAE, results indicated that the intervention's benefits were three‐fold; it further reinforced negative attitudes toward alcohol consumption during pregnancy, clarified the associated risks, and led to a significantly greater proportion of women agreeing with the UK's CMO abstinence guidance. These findings support the notion that providing information about health consequences is an effective behavioral change technique for reducing the likelihood of alcohol‐exposed pregnancies (Fergie et al., 2019). Bolstering this perspective, regression analyses revealed that those with initially more positive attitudes toward PAE and less knowledge of its risks were the most likely to experience a shift in perception postintervention. Moreover, the analyses revealed that those who had alcohol‐exposed pregnancies were more susceptible to the intervention when compared to those who did not report an alcohol‐exposed pregnancy, suggesting the intervention has some feasibility in reducing future instances of PAE. Lastly, no demographic variables emerged as significant predictors in the regression model, suggesting that the information leaflet was broadly effective across women of different ages, levels of pregnancy planning, and educational backgrounds, among other factors. Regardless, given the relative homogenous nature of the sample, this conclusion remains tentative and further research is needed to confirm these findings in more diverse populations.

Overall, educational interventions appear to show promise in shifting attitudes and improving knowledge of the risks associated with PAE within the general population. Ease of delivery, cost‐efficacy, and equitability are some of the major advantages of such interventions, which can be implemented in various formats across multiple settings. While online delivery presents significant advantages, the ability of digital public health messaging to effectively reach representative populations must be considered. Further research is needed to assess the effectiveness of such interventions across a range of cohorts, including younger women of childbearing age, individuals with substance use dependencies, and ethnic minority groups. Additionally, given the patterns of high levels of alcohol consumption prior to conception observed in multiple studies, efforts of healthcare professionals should be targeted toward women of childbearing years rather than only those who are pregnant to reduce the risk of inadvertent exposure in early pregnancy.

In a similar vein, universally applicable educational interventions that enhance public awareness of PAE and FASD could also contribute to shifts in societal norms around alcohol consumption; in turn, potentially fostering healthier drinking behaviors. The timing of such interventions may also be important, as earlier education of the risks of PAE (e.g., in secondary schools) may be more effective in driving longer‐term cultural change and in preventing future alcohol use during pregnancy. Universal preventative school‐based interventions on alcohol use among adolescents have shown small but positive effects, with potential long‐term health benefits (Strøm et al., 2014). Social determinants of health are therefore likely to be important factors impacting the efficacy of preventative educational interventions, and further research exploring these factors is warranted.

Taken together, given the intervention's low cost, ease of distribution, and alignment with current UK public health guidelines, implementing the intervention within routine care settings may be beneficial. For example, future research could assess the feasibility of distributing educational intervention leaflets during prenatal check‐up appointments, via healthcare provider partnerships. While barriers to this have been highlighted in past research, such as the lack of confidence in healthcare professionals to address PAE in expectant mothers (e.g., Schölin et al., 2021), these challenges could be mitigated via ongoing training and/or continuing professional development opportunities. Moreover, given that paperless versions of the intervention are available, another line of future work could be exploring the feasibility of embedding the online version into prenatal care smartphone applications (e.g., BadgerNotes; https://www.badgernotes.net/).

This study has several limitations. The sample primarily consisted of white, married women, with high levels of education and predominantly negative attitudes toward PAE. Moreover, 83% of women in this study reported planned pregnancies and confirmed their pregnancies within the first 6 weeks; disproportionate to national data, which indicates that over 40% of pregnancies in the UK are unplanned (Rudd et al., 2013). These factors may limit the generalizability of the findings, and the educational intervention may have had a greater impact if a more representative sample had been included. Indeed, Western, Educated, Industrialized, Rich, and Democratic (“WEIRD”) participants are often over‐represented in psychological and health research, especially in studies that recruit via self‐enrolment (de Oliveira & Baggs, 2023). While research using WEIRD populations holds value, especially given that PAE can occur across the socioeconomic spectrum (McQuire et al., 2020), the intervention's effectiveness outside of this demographic (e.g., those from socially disadvantaged or underserved communities) remains unknown.

There are several ways that future research can address this, incorporating implementation science's RE‐AIM (*Reach, Effectiveness, Adoption, Implementation, and Maintenance*) model (Glasgow et al., 2019). To enhance *Reach* for example, future research could involve approaching community and third‐sector organizations, that work closely with under‐represented populations, to aid in distributing the educational intervention. Moreover, the sample was UK‐based women, the educational intervention was administered in English, and the intervention was developed for the UK context. Thus, the utility of the intervention to wider audiences is limited. To enhance both *Effectiveness* and *Adoption*, the educational intervention could therefore be better‐adapted to be increase cultural inclusivity; in a similar manner to the original version of Project CHOICES being adapted to be culturally appropriate for use in American Indian women (Hanson et al., 2017). In the UK, one such adaptation could involve translating the intervention's content into languages spoken by minority communities (e.g., Polish, Romanian, Hindi, and Punjabi). Such efforts would help ensure that the intervention is more culturally inclusive and more widely applicable.

Secondly, educational interventions need to be developed and administered in ways that avoid reinforcing self and societal stigma toward PAE, which individuals with an alcohol‐exposed pregnancy may experience (Roozen et al., 2020). It is unclear if the educational intervention administered in this current study utilized patient and public involvement during its development. Future adaptations should actively engage individuals with lived experience of alcohol‐exposed pregnancies when developing educational tools and interventions that aim to improve the general population's knowledge of PAE and FASD. Interventions which have combined contact, including using digital methods, with an individual with a stigmatized condition and education have demonstrated attitudinal changes in the short‐ and medium‐term (Thornicroft et al., 2016). Moreover, contact interventions and education hold promise for reducing the stigma associated with PAE and FASD (Roozen et al., 2020). Additionally, adhering to the most up‐to‐date guidelines (e.g., FASD United's Language and Stigma Guide; https://fasdunited.org/stigma‐language‐guide/) during intervention development would help promote appropriate language use and in turn potentially reduce stigma.

Lastly, the assessment of the educational intervention itself may have contributed toward self and societal stigma. While care was taken to use appropriate language within the current study's questionnaire, it is possible that the statements within the Alcohol and Pregnancy Questionnaire (Peadon et al., 2011) (e.g., “The more alcohol a pregnant woman drinks, the more likely that the unborn child will be harmed”) may have elicited self‐stigma in those who had an alcohol‐exposed pregnancy at time of study involvement. While not assessed in the current study, future use of this measure should follow‐up with participants to establish if such questions can inadvertently perpetuate feelings of self‐stigma, or contribute toward societal stigma toward PAE. Additionally, while support was offered to all participants during the study debrief, further information on where to seek assistance if participants felt stigmatized should be offered in future applications of the intervention, both in clinical work and research.

This study contributes to the expanding body of research on educational interventions aimed at improving attitudes toward and knowledge of the risks associated with prenatal alcohol use. Our findings indicate that a brief, cost‐effective intervention can positively influence women's perceptions toward prenatal alcohol use and increase knowledge of its associated risks. Implementation of such interventions at both community and clinical levels in the UK could help reduce PAE, thereby lowering the risk of future cases of FASD.

## FUNDING INFORMATION

This research received no external funding.

## CONFLICT OF INTEREST STATEMENT

The authors declare no conflicts of interest.

## Supporting information


Tables S1–S4

## Data Availability

The data that support the findings of this study are available from the corresponding author upon reasonable request.
