# Supplementary material for: Knowledge as prevention: A cost‐effective intervention to reduce prenatal alcohol exposure
Source: Alcohol Clin Exp Res (Hoboken). 2025 Aug 1;49(8):1792–802. doi: 10.1111/acer.70089 (PMC12365574; doi:10.1111/acer.70089)
Supplement: Supplementary file 1 — Tables S1–S4 [file ACER-49-1792-s001.docx]

Table S1.

*Full participant characteristics.*

| **Characteristic** | **N** | **%** |
| --- | --- | --- |
| ***Age (N = 1456)*** |  |  |
| 19-25 | 90 | 6.2 |
| 26-35 | 895 | 61.5 |
| 36-50 | 471 | 32.3 |
| ***Ethnicity (N = 1468)*** |  |  |
| White (UK) | 1282 | 87.3 |
| White (Other) | 160 | 10.9 |
| Other | 26 | 1.8 |
| ***Marital status (N = 1412)*** |  |  |
| Married | 970 | 68.7 |
| In a civil partnership | 12 | .850 |
| Co-habiting | 335 | 23.7 |
| Separated | 15 | 1.1 |
| Divorced | 7 | .450 |
| Single | 73 | 5.2 |
| ***Employment status (N = 1454)*** |  |  |
| Employed full-time/Part-time | 942 | 64.8 |
| Maternity leave | 323 | 22.2 |
| Student/Unemployed/Unable to work | 189 | 13.0 |
| ***Highest qualification received (N = 1463)*** |  |  |
| Standard grades/GCSE level or Highers | 238 | 16.3 |
| Bachelor’s Degree | 516 | 35.3 |
| Masters, doctorate, or postgraduate diploma | 554 | 37.8 |
| Trade/vocational training | 106 | 7.2 |
| Other | 49 | 3.4 |
| ***Location during pregnancy (N = 1463)*** |  |  |
| Scotland | 692 | 47.3 |
| England | 555 | 37.5 |
| Wales | 115 | 7.9 |
| Northern Ireland | 101 | 6.9 |
| ***Year of pregnancy (N = 1467)*** |  |  |
| 2016 | 151 | 10.3 |
| 2017 | 152 | 10.4 |
| 2018 | 288 | 19.6 |
| 2019 | 429 | 29.2 |
| Currently pregnant (2020) | 447 | 30.5 |
| ***Pregnancy planning (N = 1467)*** |  |  |
| Planned | 1214 | 82.8 |
| Unplanned | 253 | 17.2 |
| ***Alcohol Use During Pregnancy (N = 1355)*** |  |  |
| 2-3 times per week | 7 | .500 |
| 2-4 times per month | 46 | 3.5 |
| Monthly or less | 289 | 21.3 |
| Never | 1013 | 74.7 |

Table S2.

*Cross-tab comparisons to evaluate group differences in those participants who did (n = 1120) and did not (n = 211) complete the questionnaire.*

| **Demographic Variable** | **Result** |
| --- | --- |
| Age | χ_2_ (2) = 22.47, p <.001 |
| Marital Status | χ _2_ (6) = 160.07, p <.001 |
| Educational Attainment | χ _2_ (7) = 248.19, p <.001 |
| Planned Pregnancy | χ _2_ (2) = 271.40, p <.001 |
| Year of Pregnancy | χ _2_ (5) = 267.06, p <.001 |
| Alcohol Exposed Pregnancies | χ _2_ (1) = .434, p = .510 |

Table S3.

*Comparison of pre- and post-intervention attitudes towards PAU.*

|  |  |  | **Pre-intervention** | | **Post-intervention** | |  |  |
| --- | --- | --- | --- | --- | --- | --- | --- | --- |
|  | **APQ: Attitude Items** | **N** | **M (SD)** | **Mdn** | **M (SD)** | **Mdn** | ***z*** | ***r*** |
| 1 | Pregnant women should not drink alcohol. | 1119 | 1.76 | 1.00 | 1.46 (.831) | 1.00 | -12.85^***^ | .38 |
| 2 | Pregnant women should drink less than seven units of alcohol each week. | 1118 | 1.70 (1.36) | 1.00 | 1.85 (1.47) | 1.00 | 3.85^***^ | .11 |
| 3 | *It is okay for pregnant women to drink three or four standard drinks in one day. | 1118 | 1.18 (.472) | 1.00 | 1.14 (.405) | 1.00 | -2.60^***^ | .07 |
| 4 | *It is okay for pregnant women to become intoxicated. | 1117 | 1.08 (.373) | 1.00 | 1.07 (.372) | 1.00 | .802 | .02 |
| 5 | Drinking alcohol during pregnancy can affect the unborn child. | 1119 | 1.32 (.568) | 1.00 | 1.22 (.535) | 1.00 | -6.58^***^ | .20 |
| 6 | The more alcohol a pregnant woman drinks, the more likely that the unborn child will be harmed. | 1117 | 1.24 (.541) | 1.00 | 1.16 (482) | 1.00 | -5.01^***^ | .15 |
| 7 | Drinking alcohol during pregnancy can lead to life-long disabilities in a child. | 1118 | 1.40 (.646) | 1.00 | 1.23 (.492) | 1.00 | -9.79^***^ | .29 |
| 8 | Women are aware of the effects that drinking alcohol during pregnancy can have on the unborn child. | 1118 | 2.01 (.966) | 2.00 | 2.10 (.972) | 2.00 | 4.35^***^ | .13 |
| 9 | Members of the general public are concerned about women drinking alcohol during pregnancy. | 1118 | 1.82 (.810) | 2.00 | 1.17 (.396) | 1.00 | -19.90^***^ | .59 |
| 10 | Information should be readily available to women about the effect that drinking alcohol during pregnancy may have on the unborn child. | 1118 | 1.22 (.454) | 1.00 | 1.79 (.783) | 2.00 | 18.74^***^ | .56 |
| 11 | Health professionals should ask pregnant women about how much and how often they drink alcohol. | 1119 | 1.46 (.709) | 1.00 | 1.36 (.619) | 1.00 | -7.27^***^ | .22 |
| 12 | Health professionals should advise women who are pregnant or who are thinking of becoming pregnant to give up drinking alcohol. | 1117 | 1.69 (.925) | 1.00 | 1.41 (.744) | 1.00 | -13.07^***^ | .39 |
|  | *Total ACQ: Attitudes Score* | 1105 | 17.88 (4.37) | 17.00 | 16.94 (3.91) | 16.00 | -9.72^***^ | .29 |

*Note.* Response options were 1 (strongly agree), 2 (agree), 3 (neither agree nor disagree), 4 (disagree), 5 (strongly disagree). Scores above are representative of these; for example, a median score of 2 equates to “agree”. Mdn = median; SD = standard deviation; *z* = standardised Wilcoxon signed-rank test statistic; r = Cohen’s *r*. ^*^Indicates reverse coded items. ^***^p <.001.

Table S4.

*Knowledge of effects of PAU and level of agreement with UK Chief Medical Officer’s guidance.*

|  |  | **Pre-intervention** | | **Post-intervention** | |  |  |
| --- | --- | --- | --- | --- | --- | --- | --- |
| **Knowledge** | **N** | **M**  **(SD)** | **Mdn** | **M**  **(SD)** | **Mdn** | ***z*** | ***r*** |
| PAU increases the risks of:  1. Miscarriage | 1103 | 1.91  (0.89) | 2.0 | 1.39  (0.68) | 1.0 | -18.1^***^ | .54 |
| 2. Infantile withdrawal symptoms | 1096 | 1.64  (0.77) | 1.0 | 1.43  (0.73) | 1.0 | -8.8^***^ | .27 |
| 3. Low birth weight | 1091 | 1.58  (0.67) | 1.0 | 1.33  (0.58) | 1.0 | -12.1^***^ | .37 |
| 4. Seizures | 1097 | 2.12  (0.85) | 2.0 | 1.77  (0.88) | 2.0 | -11.7^***^ | .35 |
| 5. Birth defects/malformations | 1095 | 1.77  (0.79) | 2.0 | 1.38  (0.64) | 1.0 | -15.5^***^ | .47 |
| 6. Lower IQ | 1098 | 2.08  (0.92) | 2.0 | 1.60  (0.82) | 1.0 | -17.3^***^ | .52 |
| Agreement with guidance there is no safe level | 1091 | 1.68  (1.03) | 1.0 | 1.44  (0.84) | 1.0 | -10.3^***^ | .31 |
| Agreement with guidance to abstain | 1113 | 1.69  (1.05) | 1.0 | 1.50  (0.92) | 1.0 | -9.5^***^ | .28 |
| *Total Knowledge* | 1042 | 14.53 (4.71) | 14.0 | 11.86 | 10.0 | -21.16^***^ | .65 |

*Note.* Response options were 1 (strongly agree), 2 (agree), 3 (neither agree nor disagree), 4 (disagree), 5 (strongly disagree). Scores above are representative of these; for example, a median score of 2 equates to “agree”. Mdn = median; SD = standard deviation; *z* = standardised Wilcoxon signed-rank test statistic; r = Cohen’s *r*; PAU = prenatal alcohol use. ^***^p <.001.
